# Autism risk variants in *DLG4* ortholog increase penetrance of uncommon individual behavioral trait in *Caenorhabditis elegans*

**DOI:** 10.17912/micropub.biology.001781

**Published:** 2025-10-22

**Authors:** Grace Wulffraat, Lauren Rosta, Paula Hernández, Swetha Iyer, Lisa Wang, Audrey Brumback, Jonathan Pierce

**Affiliations:** 1 Center for Learning and Memory, The University of Texas at Austin, Austin, TX, USA; 2 The University of Texas at Austin Dell Medical School, Austin, TX, USA

## Abstract

Autism is a largely neurogenetic condition characterized by atypical behaviors, including increased prevalence of motor stereotypies. We used *Caenorhabditis elegans* to model the T611I variant in the *DLG4* ortholog *dlg-1* . During phenotyping, we found that some worms intersperse typical dorsoventral swimming bends with left-right bends, resembling an orchestra conductor's arm motions. Conducting behavior occurred in 30% of wild-type but 50% of *dlg-1* mutant worms. The high proportion of conducting in *dlg-1* is recessive, rescuable with the wild-type gene, and phenocopied with another *DLG4* patient variant. This provides an example of autism variants increasing the proportion of a low-penetrant individual behavior.

**Figure 1. Autism variants in dlg-1 cause higher penetrance of the conducting pattern of swimming in C. elegans:**
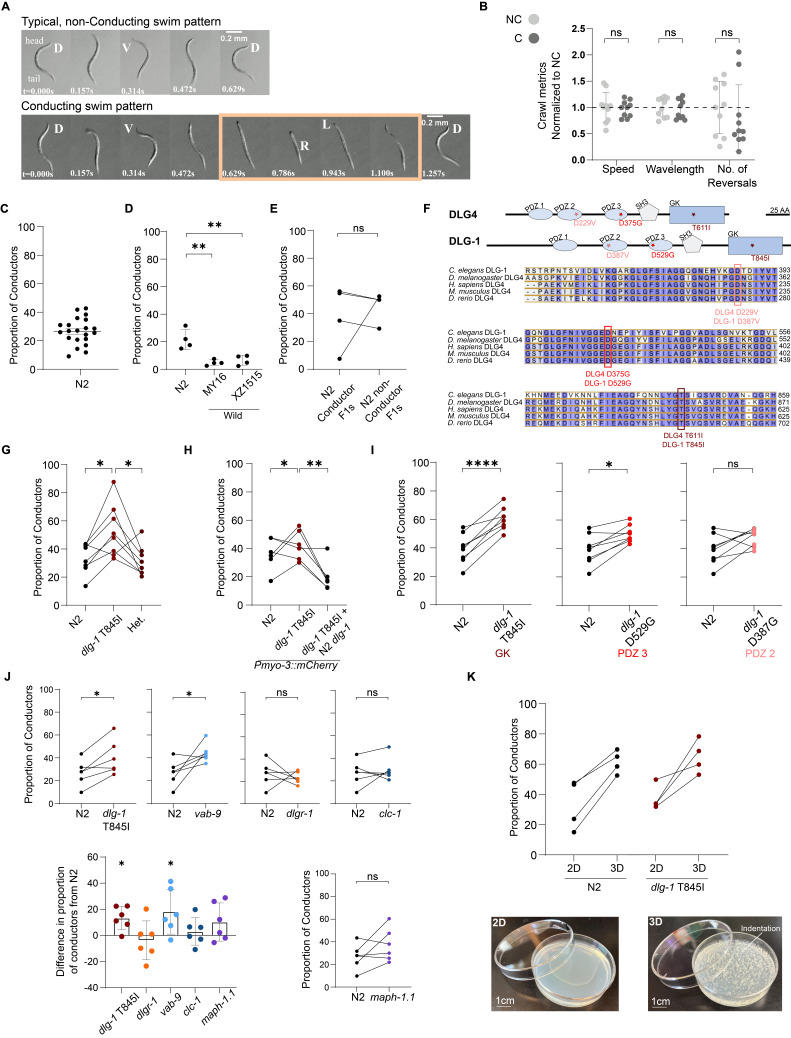
A) Typical dorsoventral pattern (top) and conducting pattern (bottom) of distinct swimming wild-type N2 worms. Dorsal (D), ventral (V), left (L), and right (R) bends. Left-right bends are highlighted in the orange box. B) Kinematic metrics of crawling are indistinguishable between non-conductor (NC) and conductor (C) wild-type N2 individuals as quantified by WormLab 2023.1.1. n=10 worms per type. C) On average, 31.6% of lab wild-type N2 populations (n=25) swim with a conducting pattern. D) Two additional wild strains, MY16 and XZ1515 , displayed conducting albeit a lower proportion than N2 based on one-way ANOVA F _(2, 9)_ =14.29 with Tukey HSD posthoc comparisons. N=4 populations per strain. E) F1 offspring of individual non-conductor and conductor parent worms display indistinguishable levels of conducting using paired t-test where t _(df=3)_ =0.982, p=0.399. N=4 populations per type. F) Conserved domains of human DLG4 and *C. elegans* DLG-1 (top). Human T611 (dark red box), D375 (red box), and D229 (light red box) are conserved across orthologs. G) *dlg-1 (T845I)* mutant populations displayed a higher proportion of conductors compared to N2 same-day controls. Heterozygous *dlg-1 (T845I* /+) populations displayed a similar proportion of conductors compared to N2 . Results compared using repeated-measures ANOVA F _(2, 21)_ =8.730 within-subjects p=0.003 with planned Tukey HSD posthoc comparisons versus *dlg-1 (T845I).* N=8 populations per strain. H) The high proportion of conducting of *dlg-1 (T845I)* was rescued to N2-like level after transformation with wild-type *dlg-1* . All strains include a red body wall reporter. Results compared with repeated-measures ANOVA F _(3, 13)_ =10.4673 within-subjects p=0.007 with planned Tukey HSD posthoc comparisons versus *dlg-1 (T845I).* N=6 populations per strain. I) *dlg-1 (T845I)* (dark red) and *dlg-1 (D529G)* (red) mutant populations display a higher proportion of conductors compared to N2 , whereas *dlg-1 (D387G)* (light red) mutant populations do not. N2 data are replotted across subpanels to show same-day comparisons with planned two-sided t-tests. Results compared with repeated-measures ANOVA F _(2, 29)_ = 17.84 within-subjects p=0.000005 with planned Dunnet's posthoc comparisons versus N2 *.* N=8 populations per strain. J) A *vab-9* deletion variant phenocopied *dlg-1 (T845I)* mutant high proportion of conducting, whereas *clc-1* , *dlgr-1* , and *maph-1.1* deletion mutants do not as evaluated with planned paired two-sided t-tests. Results compared with repeated-measures ANOVA F _(5, 24)_ = 3.926 within-subjects p=0.009 with planned Dunnet's posthoc comparisons versus N2 *.* N=6 populations per strain. K) Populations of N2 and *dlg-1 (T845I)* mutant worms grown on 3D plates (image below, right) displayed a higher proportion of conductors compared to those grown on control 2D plates (image below, left). Results compared with two-way ANOVA F _(5, 24)_ = 8.248 between-subjects p=0.003 with significant effects for dimension p<0.0005, but not genotype (p=0.488), nor genotype x dimension (p= 0.966). For all panels except B, each dot represents a population of 40-50 worms. For panel B, each dot represents an individual worm. Lines that connect dots indicate same-day paired trials. ns, not significant; * P<0.05; ** P<0.01; *** P<0.001; **** P<0.0001

## Description

Autism spectrum disorder (ASD) is a polygenic condition characterized by social difficulties, sensory processing differences, repetitive movements, and a higher proportion of low-frequency traits such as left-handedness and synesthesia (Baron-Cohen et al., 2013; Markou et al., 2017). Researchers have made progress modeling ASD, especially with mouse models of high-risk ASD gene classes such as Neurexin and Shank (Monteiro & Feng, 2017; Gomez et al., 2021). Several mouse phenotypes mimic aspects of human symptoms, including altered social behaviors, repetitive circling or grooming behaviors, and different cognitive abilities (Bryers et al., 2024). However, the hundreds of ASD-risk genes, each with many unique variants, will be challenging to model with mouse alone (e.g. https://gene.sfari.org/ ). Attributes of the nematode *C. elegans* may help surmount this challenge. Conservation of two-thirds of ASD risk genes, ease of genetic manipulation and phenotyping, and clonal nature of *C. elegans* make it a convenient model to study variants in ASD-risk genes efficiently (Kim et al. 2018).

Researchers have leveraged body morphology and behavioral phenotypes in *C. elegans* to study cellular and molecular roles of ASD-risk genes and individual patient variants (e.g. Wong et al., 2019; Cowen et al., 2024). *C. elegans* researchers have contributed greatly to our understanding of mechanisms that underlie variants in the high-risk ASD genes neuroligin and neurexin (Hu et al., 2012; Tu et al., 2015). High throughput studies have shed light on parallel pathways that are affected by groups of related ASD-risk genes (McDiarmid et al., 2020). Here, we describe a behavioral phenotype caused by ASD-risk variants in *dlg-1* , the sole *C. elegans* ortholog of human *DLG4* . The human gene is also known by aliases *PSD95* , *SAP-90* , and *SAP90* .

We observed that individual worms display distinct patterns of swimming in liquid. A majority of lab wild-type worms (strain N2 ) display the previously characterized ‘typical' swimming pattern consisting of repeated dorsoventral ‘C'-shaped bends ( Figure 1A, top) (Pierce-Shimomura et al. 2008). Alternatively, a minority display a swimming pattern that we name ‘conducting'. The worm intersperses dorsoventral bends with left-right bends, resembling the 4/4 beat hand signals of a conductor ( Figure 1A, bottom). Unlike the dorsoventral bends, which propagate to the tail and result in a characteristic C-shape, the left-right bends are prominent in the head region and do not result in a C-shaped posture. To quantify the proportion of conducting individuals, we analyzed video recordings of populations of first day adults swimming over agar for 1 minute. For the N2 strain, we found that ~30% of individuals swim with a conducting pattern and ~70% swim with a typical pattern ( Figure 1C ). Importantly, every population tested showed some conducting individuals (n = 25 populations of ~40 to 50 worms each).

Non-conducting worms swim on either their left or right side along the 2D surface of slippery agar without flipping sides. Conducting worms, however, appear to flip over after each left-right bend as evident by the alternating position of the vulva on the ventral side (15/15 times for 5 worms scored for 10 left-right bends each). The behavior appears related to the qualitatively described roll maneuver that was proposed as a strategy for the worm to reorient in 3D media ( Figure 1B and Movie S2 of Bilbao et al. 2018). We propose calling this 3D swim pattern ‘conducting' to avoid confusion with the established roller (Rol) phenotype in which mutants crawl in a circular or spiral pattern on a 2D surface and swim in a 3D corkscrew pattern in water due to the muscles detaching from the cuticle, which we did not observe in conducting worms (Brenner, 1974; Peixoto et al., 1998). By contrast, we found that conductor (C) and non-conductor (NC) worms crawl with indistinguishable speed, wavelength and reversal frequency on 2D agar as quantitatively compared using WormLab ( Figure 1B ) but display this distinct 3D conductor swim pattern in liquid.

To check if conducting behavior extended to distinct wild *C. elegans* strains, we assayed swimming in two example wild strains: MY16 from Germany and XZ1515 from Hawaii (Crombie et al., 2024). Both strains displayed conducting, but at lower penetrance than N2 ( Figure 1D ).

To test if the individual variation in conducting was explained by inheritance, we quantified the proportion of conducting in F1 progeny derived from isolated P0 individuals. We found no correlation between parent and progeny for the presence or absence of the conducting trait ( Figure 1E ).

Altogether, from the analysis of wild strains, we conclude that conducting is a natural but uncommon individual variable trait that is not influenced by its parental phenotype.

To model the ASD risk variant T611I in the human gene *DLG4* , we generated the *dlg-1 (T845I)* mutant in *C. elegans* ( Figure 1F, dark red). Note that this threonine is alternately referred to as T611 (e.g. Zhu et al., 2017) or T654 (e.g. Rodríguez-Palmero et al., 2021) in human DLG4 due to the numbering scheme for different isoforms. The T611I mutation is predicted to disrupt a guanylate kinase (GK) domain of DLG4 that is a protein-protein interaction module, not an enzyme, within the family of MAGUK synaptic scaffold proteins (Reese et al., 2007). GK has been reported to bind diverse partners including MAP1a and Disks large-associated protein 1 (DLGAP1), also known as guanylate kinase-associated protein (GKAP), but can also interact with the DLG4's own SH3 domain, which can regulate its binding to other partners (McGee and Bredt, 1999).

When phenotyping, we found that the *dlg-1 (T845I)* mutant displayed a higher proportion of conducting than N2 ( Figure 1G ). Crawling for the *dlg-1 (T845I)* mutant, however, was unchanged as determined by qualitative observations and WormLab tracking. The conducting trait appeared to be recessive and caused by the *dlg-1 (T845I)* mutation because the abnormally high proportion of conducting was reduced to wild-type N2 levels when in heterozygous form ( Figure 1G ), and was rescued by transformation with N2 genomic *dlg-1* ( Figure 1H ).

To further test the relationship between *dlg-1* and conducting, we investigated two independent DLG4 patient variants in conserved residues of DLG-1 (Rodríguez-Palmero et al. 2021). We generated two *dlg-1* mutants with mutations in distinct domains: D387V in PDZ2 and D529G in PDZ3 ( Figure 1F ). The *dlg-1 (D529G)* mutant showed a significantly higher portion of conducting compared to N2 , while *dlg-1 (D387V* ) did not ( Figure 1I ). These results strengthen evidence that *dlg-1* contributes to the conducting phenotype and suggests DLG-1 domains to explore further.

To begin to understand how variants in *dlg-1* cause a higher proportion of conducting, we investigated if mutations in related molecules phenocopy ( Figure 1J ). For human DLG4, the variant T611I was proposed to prevent binding between DLG4 and DLGAP1, which interferes with synapse development and maintenance *in vitro* (Zhu et al. 2017). We assayed the swimming of a DLGAP1 ortholog knockout mutant, *dlgr-1* , and did not observe a high conducting phenotype ( Figure 1J, orange). However, we observed a higher proportion of conducting for a *vab-9* deletion mutant ( Figure 1J, light blue). VAB-9 regulates cell adhesion in parallel with DLG-1 in *C. elegans* and is an ortholog for human *PMP22* and *TMEM47* genes (Lynch & Hardin, 2009). Notably, some variants in *PMP22* are also associated with ASD (Hurrell & Ayyash 2021; Doco-Fenzy et al. 2008). We hypothesize that high-proportion conducting may represent a convergent phenotype of ASD-associated orthologs. Future work should confirm if mutation of *vab-9* and other ASD-associated orthologs increase the proportion of conducting.

One intriguing aspect of conducting is its day-to-day variability. For wild-type N2 , populations assayed over eight months showed between 10–43% of conducting individuals ( Figure 1C ). The *dlg-1 (T845I)* mutant showed similar variability, with a remarkably high correlation for proportion of conducting between wild-type N2 and *dlg-1 (T845I)* mutant populations in paired same-day trials (R ^2^ = 0.38, 25 paired trials, p < 0.0001) The proportion of conducting did not significantly increase or decrease over the time of our study (R ^2^ = 0.158, p = 0.445). These observations show that despite the high variability, conducting behavior is a useful phenotype when same-day controls are used (e.g. five strains replotted in bar graphs relative to N2 in Figure 1J ).

The co-variation between N2 and *dlg-1 (T845I)* strains suggested that an environmental parameter influenced conducting in both strains. The environment in which worms develop can influence their behavior (e.g. Calhoun et al. 2014; Han et al. 2017; Kepler et al. 2020). Because roll maneuvers may allow worms to move efficiently in 3D space while swimming (Bilbao et al. 2018), we hypothesized that populations raised in an environment with more opportunity to move in 3D than a standard 2D agar plate may display an increased prevalence of the conducting phenotype. As predicted, we found that populations of worms raised on ‘3D plates' (2D plates poked with holes) display a higher proportion of conductors as day 1 adults ( Figure 1K ).

Overall, we conclude that environmental and genetic (via *dlg-1* ) influences on the baseline proportion of conducting individuals are independent. Future experiments will uncover potential relationships between environmental factors (e.g. surface dwelling vs. burrowed), genetics, development, plasticity, aging, and conducting behavior.

The proportion of the conducting phenotype may be useful in modeling additional ASD-associated genes and variants (Wong et al., 2019). Further, conducting behavior could facilitate the study of individual variation and plasticity, both in wild-type populations and in models of neurodevelopmental disorders (Flavell et al., 2025).

## Methods

**Strains and cultivation**

*C. elegans* were raised on nematode growth medium (NGM) plates and fed OP50 bacteria (Brenner, 1974). For the experiment depicted in Figure 1K, these standard conditions are considered 2D plates.

“3D growth plates” were made by stabbing OP50-bacteria seeded, 6-cm NGM plates with a sterile P1000 pipette tip ~200 times. Indentations varied in depth and ranged from shallow impressions to holes that perforated to the bottom of the plate. Worms were age-synchronized by bleaching and eggs were placed either on a 3D plate or a control unstabbed plate. To attempt to recover worms on the surface as well as those that borrowed in the 3D plates, we sprayed the plate at an angle with 1 mL of liquid NGM using a pipetter which appeared to encourage worms to surface. Worms were retrieved by pipette after tilting the plate. For both 2D and 3D plates, this process was repeated three times for each plate to retrieve as many worms as possible. Lastly, groups of worms from each condition were rinsed twice with liquid NGM to minimize OP50 and agar debris.

JPS1867 and JPS1868 strains were generated by microinjection with 5 ng/µL of a *Pmyo-3::mCherry* red body wall (RBW) (pCFJ104). Wild-type *dlg-1* was amplified from N2 via PCR with primers GATCTCCAACACTGTGTCGC and GGAAGCACATTTCGAAACGG which included 2375 bp and 2071 bp of 5' and 3' UTR, respectively. JPS1879 strain was generated by transforming strain PHX8797 with 5 ng/µL RBW and 10 ng/µL N2 *dlg-1* .

SUNY Biotech used CRISPR/Cas9 to generate strain PHX8797 with a T845I missense mutation in *dlg-1* in an N2 background from: **GCG** GGACAATTCCAAAACAAT **CTC** TACGGA **ACT** AGCATTCAAAGCGTCCGAGATGTC **GCCAAC** , to:

**GCC** GGACAATTCCAAAACAAT **tTg** TACGGA **AtT** AGCATTCAAAGCGTCCGAGATGTC **GCGAAt.**

InVivo Biosystems used CRISPR/Cas12a with homology-directed repair (HDR) as described in Paix et al (2015) to generate two strains with missense mutations in *dlg-1* in an N2 background. The *dpy-10* co-CRISPR methods described in Arribere et al (2014) was used to identify animals in which the CRISPR/Cas12a system was active.

First, strain JPS1891 with a D387V missense mutation used:

5' Cas12a sgRNA sequence: 5' - CTTGACCACCAGCAATAGAGAAA - 3'

3' Cas12a sgRNA sequence: 5' - GTCACATAGATATCGGTATCTCC - 3'

ssODN: 5'-CTTTTTTTAGGGAGCACGTGGACTTGGTTTCTCTATCGCCGGCGGCCAGGGCAATGAACATGTCAAGGGGGTCACTGATATCTATGTGACGAAAATCATTGAGGAGGGAG - 3'

Second, strain JPS1893 with a D529G missense mutation used:

5' Cas9 sgRNA sequence: 5' - TGTACTGGACGGGGTTCGAG - 3'

3' Cas9 sgRNA sequence: 5' - GGAGGTGTTGCTGATCTTAG - 3'

ssODN: 5'-GCTACAGTTCACAAGCCCCCATCGCAATTCCACTCGAGCCAAGACCAGTTCAGTTGGTTAAGGGACAGAACGGACTCGGCTTTAATATCGTCGGAGGAGAGGGAAACGAACCAATTTATATCTCCTTCGTCCTTCCTGGCGGAGTCGCCGACCTTAGTGGAAACGTGAAGACTGGAGACGTTCTTCTTG - 3'

Missense mutations above were verified as homozygous after sequencing *dlg-1* from singled worms across three generations with the following primers: JPIE03F2 AGCCAACAATTCATGCCAACT, JPIE03R2 AGCAACAGAGTTTTCACGTGAT, JPIE02_seq_For GCA ATTCATTCTCCATCGGCT, JPIE02_seq_Rev GCGGTTGTAGTCTTGAACGG

**Behavioral analyses**

*Crawling* : Crawling and body morphology was quantified with WormLab 2023.1.1 (MBF Bioscience, USA).

*Swimming* : For most experiments, worms were age-synchronized by 5-hour clutches of laid eggs. Approximately 50, day-1 adult individuals were picked into either 1) 1.5 mL liquid NGM on an NGM agar plate (figures 1G and 1H), or 2) 1 mL liquid NGM in one well of a flat-bottom clear 24-well plastic plate (figures 1D, 1E, 1I, and 1J).

Swimming behavior was recorded for 1 minute at 30 Hz for groups of ~50 worms after a 10-minute acclimation period. Each individual was scored blind to genotype and culture conditions. Individuals were classified as conductors if they performed 3 or more left-right bends within a period of ~21 dorsoventral bends. All other individuals were classified as non-conductors.

**Statistical Analysis**

Planned Student's t-tests were used to evaluate differences in genotypes paired by same-day trials (Microsoft Excel Version 2507). Repeated measures ANOVAs were used to compare proportion of conductor worms in same-day trials across genotypes (SPSS version 29.0.2.0 (20)). Two-way ANOVA was used to compare the main effect and the interactions between dimension and genotype variables. Tukey or Dunnet's posthoc tests were used to compare individual groups with planned tests.

For experiments in Fig 1I, for each N2 negative control and *dlg-1 (T845I)* positive control trial, two *dlg-1 (D529G)* and *dlg-1 (D387G)* paired trials were conducted. The values for each set of two trials were averaged and considered a single value to be compared with the paired negative and positive controls.

## Reagents

**Table:** 

**Strain**	**Genotype**	**Originated from**
N2	*Caenorhabditis elegans* WT	CGC
PHX8797	*dlg-1 (syb8797[T845I]) *	SUNY Biotech
JPS1867	Red Body Wall (RBW) control = N2 *vxEx2050[Pmyo-3::mCherry]*	JPS lab, this study
JPS1868	RBW *dlg-1 (syb8797[T845I])* control = PHX8797 *vxEx2051[Pmyo-3::mCherry]*	JPS lab, this study
JPS1879	Rescue strain = *dlg-1 (T845I) vxEx2052[P dlg-1 :: dlg-1 :: dlg-1 UTR + Pmyo-3::mCherry] *	JPS lab, this study
FM1176	*dlgr-1* knock-out deletion strain = *ltSi1412 [pNA20; Pmex-5::mNeonGreen:: tbb-2 operon linker mCh:: his-11 ::P tbb-2 ; cb- unc-119 (+)]I; dlgr-1 (syb8768) III; ltIs44 [pAA173; pie-1p-mCh::PH(PLC1delta1) + unc-119 (+)]* V	McNally Lab, described in Mahantesh Magadum &, McNally, 2024
QQ258	*vab-9 ( ju6 )* II. partial deletion	CGC, described in Simske JS, et al. Nat Cell Biol. 2003 Jul;5(7):619-25.
VC4576	*maph-1.1* knock-out deletion strain = *maph-1.1 ( gk5647 [loxP + myo-2p::GFP:: unc-54 3' UTR + rps-27p::neoR:: unc-54 3' UTR + loxP])* I.	CGC, described in Au et al., G3 9(1): 135-144 2019
VC4795	*clc-1* knock-out deletion = *clc-1 ( gk5863 [loxP + myo-2p::GFP:: unc-54 3' UTR + rps-27p::neoR:: unc-54 3' UTR + loxP])* X.	CGC, described in Au et al., G3 9(1): 135-144 2019
MY16	Wild strain from Germany	CaeNDR, described in Crombie et al., 2024
XZ1515	Wild strain from Hawaii	CaeNDR, described in Crombie et al., 2024
JPS1891	*dlg-1 (vx2053[D387V]) *	JPS lab via InVivo Biosystems, this study
JPS1893	*dlg-1 (vx2054[D529G)] *	JPS lab via InVivo Biosystems, this study

## Data Availability

Description: Example WT N2 conductor swimming. Resource Type: Audiovisual. DOI: https://doi.org/10.22002/hsggq-6xm74 Description: Example WT N2 non-conductor swimming. Resource Type: Audiovisual. DOI: https://doi.org/10.22002/31znh-6fh10

## References

[R1] Arribere JA, Bell RT, Fu BX, Artiles KL, Hartman PS, Fire AZ (2014). Efficient marker-free recovery of custom genetic modifications with CRISPR/Cas9 in Caenorhabditis elegans.. Genetics.

[R2] Baron-Cohen S, Johnson D, Asher J, Wheelwright S, Fisher SE, Gregersen PK, Allison C (2013). Is synaesthesia more common in autism?. Mol Autism.

[R3] Bilbao A, Patel AK, Rahman M, Vanapalli SA, Blawzdziewicz J (2018). Roll maneuvers are essential for active reorientation of Caenorhabditis elegans in 3D media.. Proc Natl Acad Sci U S A.

[R4] Brenner S (1974). The genetics of Caenorhabditis elegans.. Genetics.

[R5] Bryers A, Hawkes CA, Parkin E, Dawson N (2024). Progress towards understanding risk factor mechanisms in the development of autism spectrum disorders.. Biochem Soc Trans.

[R6] Calhoun AJ, Chalasani SH, Sharpee TO (2014). Maximally informative foraging by Caenorhabditis elegans.. Elife.

[R7] Cowen MH, Haskell D, Zoga K, Reddy KC, Chalasani SH, Hart MP (2024). Conserved autism-associated genes tune social feeding behavior in C. elegans.. Nat Commun.

[R8] Crombie TA, McKeown R, Moya ND, Evans KS, Widmayer SJ, LaGrassa V, Roman N, Tursunova O, Zhang G, Gibson SB, Buchanan CM, Roberto NM, Vieira R, Tanny RE, Andersen EC (2024). CaeNDR, the Caenorhabditis Natural Diversity Resource.. Nucleic Acids Res.

[R9] Doco-Fenzy M, Holder-Espinasse M, Bieth E, Magdelaine C, Vincent MC, Khoury M, Andrieux J, Zhang F, Lupski JR, Klink R, Schneider A, Goze-Martineau O, Cuisset JM, Vallee L, Manouvrier-Hanu S, Gaillard D, de Martinville B (2008). The clinical spectrum associated with a chromosome 17 short arm proximal duplication (dup 17p11.2) in three patients.. Am J Med Genet A.

[R10] Flavell SW, Oren-Suissa M, Stern S (2025). Sources of behavioral variability in C.&nbsp;elegans: Sex differences, individuality, and internal states.. Curr Opin Neurobiol.

[R11] Gomez AM, Traunmüller L, Scheiffele P (2021). Neurexins: molecular codes for shaping neuronal synapses.. Nat Rev Neurosci.

[R12] Han B, Dong Y, Zhang L, Liu Y, Rabinowitch I, Bai J (2017). Dopamine signaling tunes spatial pattern selectivity in C. elegans.. Elife.

[R13] Hu Z, Hom S, Kudze T, Tong XJ, Choi S, Aramuni G, Zhang W, Kaplan JM (2012). Neurexin and neuroligin mediate retrograde synaptic inhibition in C. elegans.. Science.

[R14] Hurrell S, Ayyash H - 433 A patient with autistic spectrum disorder and 17p12 duplication – literature review: BMJ Paediatrics Open 2021;5.

[R15] Kepler LD, McDiarmid TA, Rankin CH (2020). Habituation in high-throughput genetic model organisms as a tool to investigate the mechanisms of neurodevelopmental disorders.. Neurobiol Learn Mem.

[R16] Kim W, Underwood RS, Greenwald I, Shaye DD (2018). OrthoList 2: A New Comparative Genomic Analysis of Human and
*Caenorhabditis elegans*
Genes.. Genetics.

[R17] Lynch AM, Hardin J (2009). The assembly and maintenance of epithelial junctions in C. elegans.. Front Biosci (Landmark Ed).

[R18] Mahantesh Magadum M, McNally F (2024). DLGR-1, a homolog of vertebrate DLGAP proteins, regulates spindle length and anaphase velocity during C. elegans meiosis.. MicroPubl Biol.

[R19] Markou P, Ahtam B, Papadatou-Pastou M (2017). Elevated Levels of Atypical Handedness in Autism: Meta-Analyses.. Neuropsychol Rev.

[R20] McDiarmid TA, Belmadani M, Liang J, Meili F, Mathews EA, Mullen GP, Hendi A, Wong WR, Rand JB, Mizumoto K, Haas K, Pavlidis P, Rankin CH (2019). Systematic phenomics analysis of autism-associated genes reveals parallel networks underlying reversible impairments in habituation.. Proc Natl Acad Sci U S A.

[R21] McGee AW, Bredt DS (1999). Identification of an intramolecular interaction between the SH3 and guanylate kinase domains of PSD-95.. J Biol Chem.

[R22] Monteiro P, Feng G (2017). SHANK proteins: roles at the synapse and in autism spectrum disorder.. Nat Rev Neurosci.

[R23] Paix A, Folkmann A, Rasoloson D, Seydoux G (2015). High Efficiency, Homology-Directed Genome Editing in Caenorhabditis elegans Using CRISPR-Cas9 Ribonucleoprotein Complexes.. Genetics.

[R24] Peixoto CA, de Melo JV, Kramer JM, de Souza W (1998). Ultrastructural analyses of the Caenorhabditis elegans rol-6 (su1006) mutant, which produces abnormal cuticle collagen.. J Parasitol.

[R25] Pierce-Shimomura JT, Chen BL, Mun JJ, Ho R, Sarkis R, McIntire SL (2008). Genetic analysis of crawling and swimming locomotory patterns in C. elegans.. Proc Natl Acad Sci U S A.

[R26] Reese ML, Dakoji S, Bredt DS, Dötsch V (2007). The guanylate kinase domain of the MAGUK PSD-95 binds dynamically to a conserved motif in MAP1a.. Nat Struct Mol Biol.

[R27] Rodríguez-Palmero A, Boerrigter MM, Gómez-Andrés D, Aldinger KA, Marcos-Alcalde Í, Popp B, Everman DB, Lovgren AK, Arpin S, Bahrambeigi V, Beunders G, Bisgaard AM, Bjerregaard VA, Bruel AL, Challman TD, Cogné B, Coubes C, de Man SA, Denommé-Pichon AS, Dye TJ, Elmslie F, Feuk L, García-Miñaúr S, Gertler T, Giorgio E, Gruchy N, Haack TB, Haldeman-Englert CR, Haukanes BI, Hoyer J, Hurst ACE, Isidor B, Soller MJ, Kushary S, Kvarnung M, Landau YE, Leppig KA, Lindstrand A, Kleinendorst L, MacKenzie A, Mandrile G, Mendelsohn BA, Moghadasi S, Morton JE, Moutton S, Müller AJ, O'Leary M, Pacio-Míguez M, Palomares-Bralo M, Parikh S, Pfundt R, Pode-Shakked B, Rauch A, Repnikova E, Revah-Politi A, Ross MJ, Ruivenkamp CAL, Sarrazin E, Savatt JM, Schlüter A, Schönewolf-Greulich B, Shad Z, Shaw-Smith C, Shieh JT, Shohat M, Spranger S, Thiese H, Mau-Them FT, van Bon B, van de Burgt I, van de Laar IMBH, van Drie E, van Haelst MM, van Ravenswaaij-Arts CM, Verdura E, Vitobello A, Waldmüller S, Whiting S, Zweier C, Prada CE, de Vries BBA, Dobyns WB, Reiter SF, Gómez-Puertas P, Pujol A, Tümer Z (2021). DLG4-related synaptopathy: a new rare brain disorder.. Genet Med.

[R28] Tu H, Pinan-Lucarré B, Ji T, Jospin M, Bessereau JL (2015). C. elegans Punctin Clusters GABA(A) Receptors via Neuroligin Binding and UNC-40/DCC Recruitment.. Neuron.

[R29] Wong WR, Brugman KI, Maher S, Oh JY, Howe K, Kato M, Sternberg PW (2019). Autism-associated missense genetic variants impact locomotion and neurodevelopment in Caenorhabditis elegans.. Hum Mol Genet.

[R30] Zhu J, Zhou Q, Shang Y, Li H, Peng M, Ke X, Weng Z, Zhang R, Huang X, Li SSC, Feng G, Lu Y, Zhang M (2017). Synaptic Targeting and Function of SAPAPs Mediated by Phosphorylation-Dependent Binding to PSD-95 MAGUKs.. Cell Rep.

